# Diagnostic Accuracy of Artificial Intelligence Versus Musculoskeletal Radiologists for Foot and Ankle Fracture Detection on Radiographs

**DOI:** 10.3390/diagnostics16162507

**Published:** 2026-08-09

**Authors:** David Ferreira Branco, Paul Botti, Hicham Bouredoucen, Quentin Pedrini, Bilal Abs, Nicolas Berla, Pierre-Alexandre Poletti, Alexandra Platon, Sana Boudabbous

**Affiliations:** Department of Radiology, Geneva University Hospitals, 1205 Geneva, Switzerland; paul.botti@hug.ch (P.B.); hicham.bouredoucen@hug.ch (H.B.); quentin.pedrini@hug.ch (Q.P.); bilal.abs@hug.ch (B.A.); nicolas.berla@hug.ch (N.B.); pierre-alexandre.poletti@hug.ch (P.-A.P.); alexandra.platon@hug.ch (A.P.); sana.boudabbous@hug.ch (S.B.)

**Keywords:** foot, ankle, fracture detection, artificial intelligence, radiography

## Abstract

**Background/Objectives:** To evaluate the diagnostic accuracy of a standalone artificial intelligence fracture detection software on foot and ankle radiographs, compared with board-certified musculoskeletal radiologists, with emphasis on midfoot injuries. **Materials and Methods:** This retrospective single-center diagnostic accuracy study included adult patients presenting to the emergency department with acute foot or ankle trauma. Radiographs were interpreted in real time, analyzed retrospectively by musculoskeletal (MSK) radiologists during the routine workflow and subsequently analyzed independently by an artificial intelligence system. The reference standard was structured clinical follow-up, complemented by cross-sectional imaging when the radiograph was equivocal. Diagnostic performance metrics and inter-reader agreement were calculated. **Results:** A total of 701 examinations (mean age, 42 ± 17 years; 376 men) were included; 319 fractures were identified, including 29 Chopart and 26 Lisfranc injuries. Overall sensitivity, specificity, and accuracy were 74.3%, 83.0%, and 79.0% for artificial intelligence, 84.0%, 95.5%, and 90.3% for radiologists, respectively (*p* < 0.0001; κ = 0.65). For Chopart and Lisfranc fractures, radiologists demonstrated higher sensitivity, while specificity remained excellent for both approaches, but the paired comparisons did not reach significance. **Conclusions:** Standalone artificial intelligence achieved high diagnostic performance for foot and ankle fracture detection on radiographs but was less sensitive than MSK radiologists for complex midfoot injuries.

## 1. Introduction

Trauma to the foot and ankle is a frequent cause of visits to the emergency department and often requires imaging to confirm or exclude fractures. Standard radiography (XR) remains the primary modality due to its accessibility and speed. However, it often lacks sensitivity, particularly in anatomically complex regions such as the midfoot. Fractures involving the Chopart, which includes the talonavicular and calcaneocuboid articulations, are among the most frequently missed on initial imaging due to overlapping bony structures and subtle findings [1,2]. Similarly, Lisfranc (tarsometatarsal) injuries are frequently underdiagnosed on conventional radiographs [3] particularly when the pattern is subtle or predominantly ligamentous avulsion. Optimized projections and weight-bearing acquisitions can improve visualization of the second tarsometatarsal joint and unmask dynamic instability [3], thereby increasing diagnostic yield. Beyond optimized radiographs, cross-sectional imaging, particularly CT [4], demonstrates higher diagnostic performance for surgically confirmed subtle Lisfranc injuries. In this same study, MRI demonstrated slightly higher overall diagnostic accuracy than CT, particularly for injuries involving the second and cuneiform rays, while CT remained comparable or superior for the first ray. However, the limited availability of MRI in emergency settings continues to restrict its routine use in the acute evaluation of foot trauma.

Up to 30–40% of midfoot fractures are initially overlooked on plain radiographs, which can lead to a delayed diagnosis, functional impairment or post-traumatic arthropathy [4,5,6]. Cone-beam CT (CBCT) improves diagnostic performance in these cases and is a valuable reference tool when available [1]. However, CBCT is not routinely performed in all institutions, and, in many cases, clinical evaluation remains the de facto standard for final diagnosis.

Artificial intelligence (AI) systems have emerged as promising tools for automatically detecting fractures on limb radiographs. Several recent studies conducted in emergency settings have evaluated commercially available AI software and shown that it can improve reader sensitivity and reduce missed diagnoses, particularly for subtle or non-displaced fractures that are frequently overlooked under time pressure [7,8,9]. Reported benefits include higher detection sensitivity when AI outputs are integrated into the reading workflow, a reduction in diagnostic discrepancies, and shorter reporting times, although the magnitude of the clinical impact has varied across studies and workflows [7,8,9]. Importantly, this evidence is almost entirely retrospective; prospective clinical validation of these tools remains scarce. Moreover, while the performance of such algorithms has been validated across various skeletal regions, their accuracy in detecting complex midfoot injuries, particularly Lisfranc and Chopart joint injuries, remains under-investigated.

Hence, our study aims to compare the diagnostic performance (sensitivity, specificity and accuracy) of AI-based fracture detection software (BoneView™) compared to board-certified musculoskeletal radiologists’ reports on routine radiographs for foot and ankle fractures. Special emphasis is placed on a subgroup analysis of Chopart and Lisfranc joint fractures. The reference standard was structured clinical follow-up, complemented by cross-sectional imaging (CBCT) when the radiograph was equivocal.

## 2. Materials and Methods

### 2.1. Study Design and Population

**Scheme 1:**
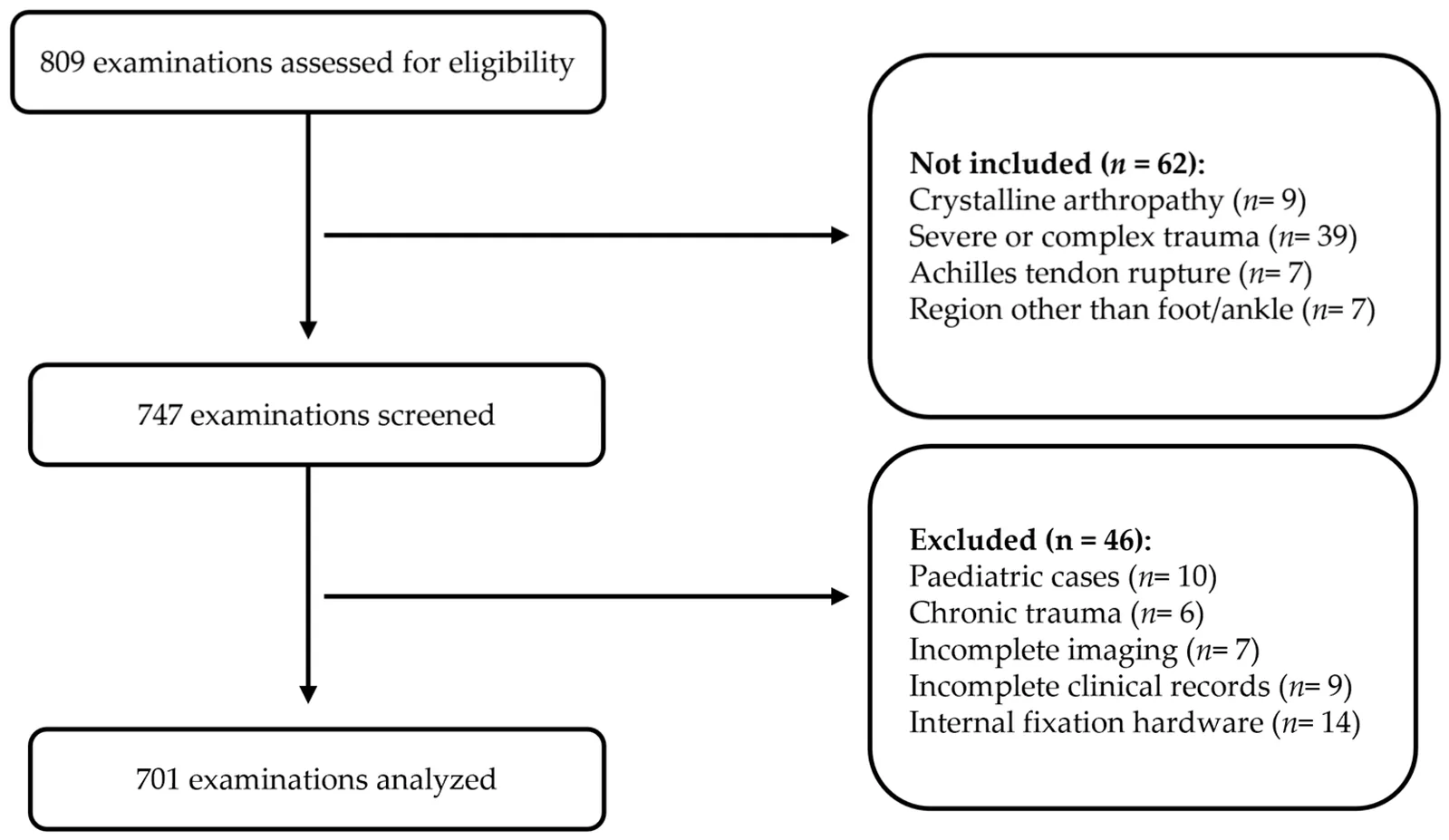
STARD flow diagram of examination selection.

This retrospective, single-center diagnostic performance study was conducted at a tertiary care hospital. The protocol for this study was approved by the cantonal research ethics committee (CCER 2020-02812), which waived patients’ informed consent. Over a 3-month period (January to March 2023), the radiographs of all consecutive patient examinations (*n* = 747) who presented to the emergency department with acute foot or ankle trauma and underwent standard radiography within 24 h of injury were included (see Scheme 1).

Patients were eligible if they had radiographs of the foot and/or ankle performed within the specified timeframe and if a final diagnosis regarding the presence or absence of a fracture could be established. In patients with a normal or equivocal radiograph, the reference standard was structured prospective clinical follow-up for up to 6 weeks through a dedicated ambulatory emergency trauma clinic, based principally on clinical evolution, complemented by cross-sectional imaging when the radiograph was equivocal. This clinical reference was established independently of the musculoskeletal radiology report evaluated in this study. Radiographs were interpreted in real time during routine clinical workflow, analyzed retrospectively by board-certified musculoskeletal radiologists as part of routine clinical emergency workflow, with subsequent anonymization of images for standalone artificial intelligence evaluation.

Exclusion criteria included pediatric examinations (*n* = 10), follow-up or chronic trauma cases (*n* = 6), incomplete imaging (*n* = 7) or clinical records (*n* = 9), and the presence of internal fixation hardware at the injury site (*n* = 14). Examinations with complex or severe injuries, displaced fractures or high-energy trauma, notably with hindfoot involvement, were not included (*n* = 39) (see flow chart, Scheme 1).

This inclusion strategy reflects the limited sensitivity of radiographs for midfoot injuries and the role of cross-sectional imaging as the reference standard when available [5,6].

### 2.2. Imaging Protocol and Interpretation

All radiographs were acquired using standard digital imaging protocols. Depending on clinical suspicion, views included posteroanterior, oblique, and lateral projections of the ankle, including the midfoot. When a Lisfranc or midfoot injury was suspected, oblique and posteroanterior weight-bearing radiographs of the forefoot were systematically obtained. Radiographs had been previously interpreted during routine clinical workflow. For this retrospective study, images were subsequently anonymized and exported to a dedicated research PACS for standalone AI analysis, fully independent of the clinical report. Fracture detection was performed with BoneView (Gleamer, Paris, France; version 2.5.1, June 2025), an FDA-cleared (510(k) K212365) and CE-marked computer-assisted fracture detection device; the foot and ankle are within its cleared indications for use (in adults). The algorithm was developed on external multicenter datasets and was not trained on data from our institution. The present cohort constitutes a fully external validation. BoneView provides two operating points: a high-sensitivity point (DOUBT FRACT, dotted bounding box) and a high-specificity point (FRACT, solid bounding box). Because missing a fracture is the primary concern in emergency trauma, an examination was considered AI-positive at the high-sensitivity operating point (DOUBT FRACT). Any suspicious or subtle finding flagged by the algorithm was therefore counted as positive. The AI analyzed each radiographic view independently, and an examination was classified as AI-positive if a fracture was flagged on at least one acquired view. Although BoneView is labeled as a concurrent reading aid, it was evaluated in standalone mode, processing the anonymized DICOM [10,11,12]. All AI results were stored in a separate research PACS for subsequent comparison with the radiologists’ clinical reports.

For patients undergoing CBCT, it was performed using a dedicated system (OnSight, Carestream Health, Rochester, New York, USA), with the same protocol, featuring a 58 cm gantry aperture and a mobile patient table with a standard protocol published in a previous study [1]. Each acquisition included anteroposterior and anterolateral scout views. Weight-bearing CBCT was obtained whenever clinically feasible. Images were reconstructed in both coronal and sagittal planes using a first-generation model-based iterative reconstruction algorithm, with a bone kernel and a slice thickness of 0.26 mm. Bone window settings were standardized (window width: 1500 HU; window level: 300 HU [1]).

The final analysis compared the radiologist’s clinical reports (issued during routine emergency workflow) with the AI standalone outputs, both assessed against the hierarchical reference standard. This pragmatic reference, structured clinical follow-up complemented by cross-sectional imaging when the radiograph was equivocal, reflects real-world emergency practice [7,8].

For the purposes of this study, fractures were grouped into three categories: Chopart injuries, Lisfranc injuries, and other locations involving any remaining foot or ankle regions.

### 2.3. Statistical Analysis

Diagnostic performance was assessed for both the radiologist and the AI software in terms of sensitivity, specificity, accuracy, positive predictive value (PPV), and negative predictive value (NPV), using the hierarchical reference standard. Agreement between the AI and the radiologist was quantified using Cohen’s kappa coefficient. Because both readers were applied to the same examinations, paired comparisons were used. Sensitivity and specificity were compared with a McNemar test stratified on reference-standard status; overall accuracy was compared with McNemar on the correct/incorrect classification (exact binomial McNemar when discordant counts were small). No correction for multiple comparisons was applied. All statistical analyses were conducted using Medcalc software (MedCalc Software Ltd., Ostend, Belgium, version 23.3.7 August 2025), with significance set at *p* < 0.05. This study was conducted and reported in accordance with the STARD 2015 and STARD-AI recommendations for diagnostic accuracy studies involving artificial intelligence.

## 3. Results

### 3.1. Descriptive Results

In total, 701 radiologic examinations were analyzed, comprising 376 men and 325 women (mean age 42.0 ± 17.5 years; range 10–99; 95% CI 40.7–43.3). Overall, 45.5% of cases had a fracture, and 54.5% had no fracture; among fractures, other locations predominated (82.8%; *n* = 264), with Chopart and Lisfranc injuries representing 9.1% (*n* = 29) and 8.1% (*n* = 26), respectively. The hierarchical reference standard relied chiefly on clinical assessment in 84.2% (*n* = 590) of cases, with CBCT available in 15.8% (*n* = 111). All analyses were performed at the examination level, with the radiologists’ routine clinical reports considered as a single reference reader.

### 3.2. Overall Accuracy Results

**Figure 1:**
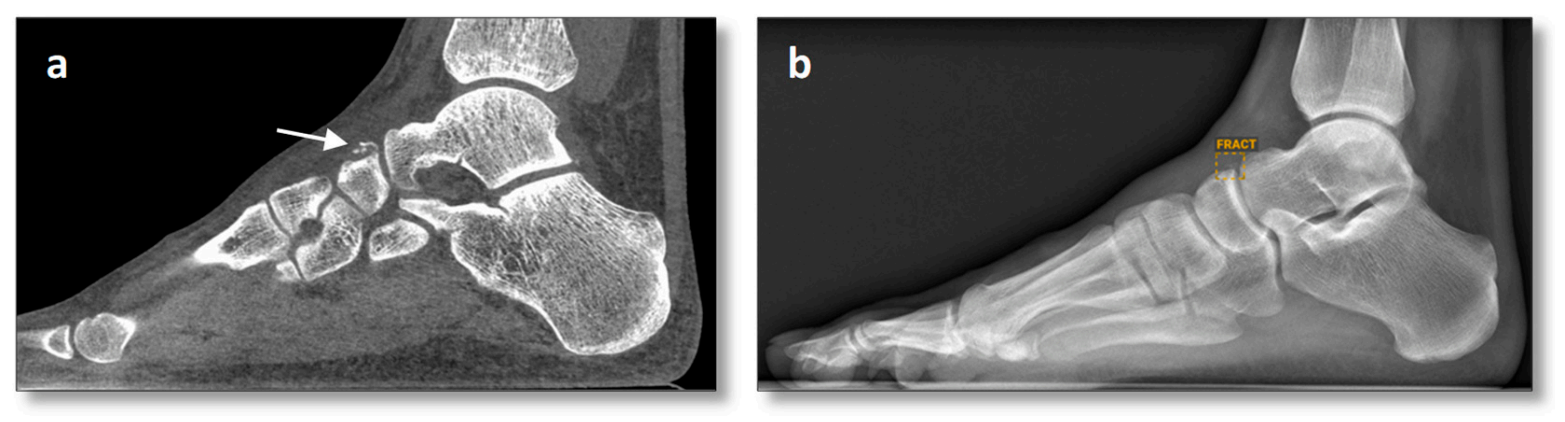
Concordant case of a dorsal talonavicular joint injury visible on cone-beam CT (arrow) (**a**), detected on radiographs both by the radiologist and the autonomous commercial AI (orange dotted bounding box) (**b**).

Compared with the reference gold standard, the MSK radiologists and the AI system demonstrated high overall diagnostic performance, with accuracy values of 90.3% (268 true positives (TPs); 51 false negatives (FNs); 17 false positives (FPs); 365 true negatives (TNs)) and 79.0% (237 TPs;82 FNs; 65 FPs; 317 TNs), respectively (confusion matrices for both readers, overall and per subgroup, are provided in Table 1). Sensitivity and specificity were 84.0% and 95.5% for the radiologist, and 74.3% and 83.0% for the AI system. Agreement between the methods was substantial (κ = 0.65) (see Figure 1). The radiologist showed significantly higher sensitivity, specificity and accuracy than standalone AI (all *p* < 0.0001) (see Table 2). Because the fracture prevalence in this cohort (45.5%) was higher than in most emergency populations, PPV and NPV are setting-specific and should not be extrapolated; prevalence-independent likelihood ratios are therefore also reported (Table 2).

### 3.3. Subgroup Results

In the Chopart subgroup, both approaches showed very high specificity (radiologist 99.7% 670 TNs, AI 99.6% 669 TNs) with no significant difference (Exact McNemar *p* = 1.0). The sensitivity differed substantially, with the radiologist identifying 24 TPs and 5 FNs (82.8%), while the AI system identified 18 TPs with 11 FNs (62.1%) (see Table 1), but this difference did not reach significance (exact McNemar *p* = 0.07). Overall accuracy reached 99.0% for the radiologist and 98.0% for AI, a difference that was statistically significant (exact McNemar *p* = 0.039) with near-perfect agreement (κ = 0.80) (see Table 3).

FNs by AI (*n* = 11) mainly involved subtle cortical disruptions at the calcaneocuboid joint or minimally displaced talonavicular fractures, often masked by overlapping bony contours. A few isolated FPs (*n* = 3) were generated by the AI in areas of degenerative change or vascular channels misinterpreted as fracture lines.

In the Lisfranc subgroup, radiologists recorded 21 TPs, 5 FNs, 0 FPs, and 675 TNs, whereas the AI system produced 17 TPs, 9 FNs, 1 FP, and 674 TNs (Table 1 and Table 4). Based on these values, both approaches demonstrated excellent specificity (100.0% for radiologists and 99.9% for AI), while sensitivity reached 80.8% for radiologists and 65.4% for the AI system. Accuracy was similarly high for both readers, measured at 99.3% for radiologists and 98.6% for AI, without significant differences (exact McNemar specificity *p* = 1.0, sensitivity *p* = 0.22, accuracy *p* = 0.125). Agreement between methods was almost perfect (κ = 0.82).

In this subgroup, AI FNs (*n* = 9) corresponded predominantly to subtle diastases of the first or second tarsometatarsal joints without clear cortical break (*n* = 5), whereas the single AI FP occurred in a case of mild joint malalignment interpreted as fracture.

When all fracture locations other than Chopart and Lisfranc were grouped together, radiologists significantly outperformed standalone AI for sensitivity (84.5% (79.6–88.3) vs. 76.5% (71.0–81.2); *p* = 0.0023), specificity (96.0% (93.6–97.6) vs. 83.9% (79.8–87.2); *p* < 0.0001) and accuracy (91.3% (88.8–93.2) vs. 80.8% (77.6–83.7); *p* < 0.0001).

## 4. Discussion

In our emergency cohort, both the board-certified musculoskeletal radiologists and the standalone commercial AI algorithm demonstrated high diagnostic performance for fracture detection on foot and ankle radiographs. The radiologists showed higher sensitivity, specificity and accuracy (*p* < 0.0001), a pattern consistent with previous evaluations of autonomous AI tools in appendicular trauma [7,8,9]. This pattern mirrors prior head-to-head evaluations directly comparing autonomous AI systems with human readers. Across these studies, standalone AI generally achieved diagnostic performance comparable to that of general radiologists but remained below MSK experts, particularly for anatomically complex or subtle injuries [7,8,9]. Our findings therefore align with existing evidence, reinforcing that, as currently configured and deployed in autonomous mode, AI serves best as a complementary tool within emergency imaging workflows.

A distinctive aspect of our study lies in the analysis of midfoot fractures, particularly Chopart and Lisfranc injuries, regions for which AI performance has been minimally documented to date.

**Figure 2:**
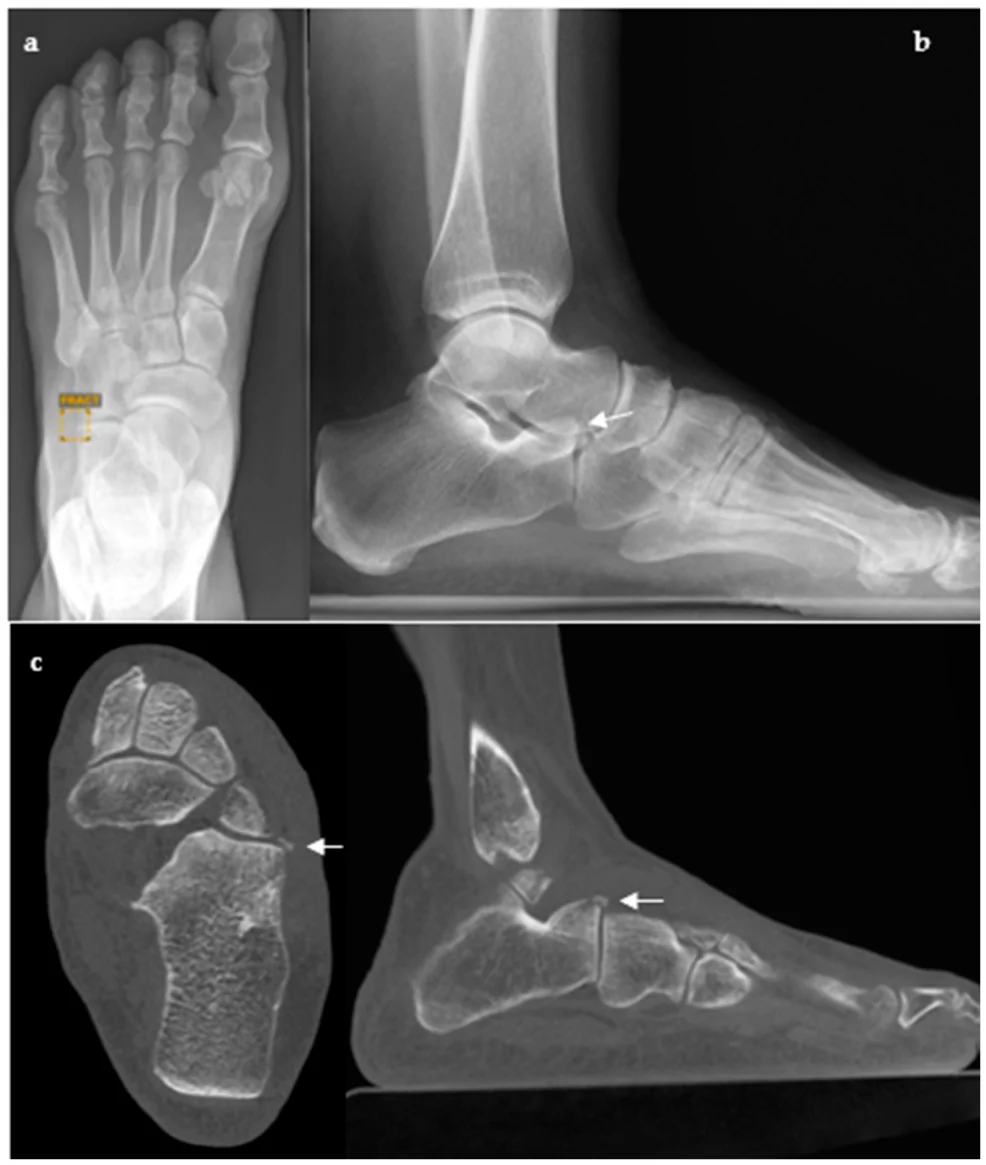
Discordant case involving Chopart joints. The autonomous AI identified part of the injury: it detected the calcaneocuboid fracture (orange dotted bounding box: FRACT) on the axial view (**a**) and missed the avulsion of the calcaneal rostrum (white arrow) on the lateral view (**b**). This fracture was correctly identified by the radiologist and confirmed by CBCT (white arrow) (**c**).

**Figure 3:**
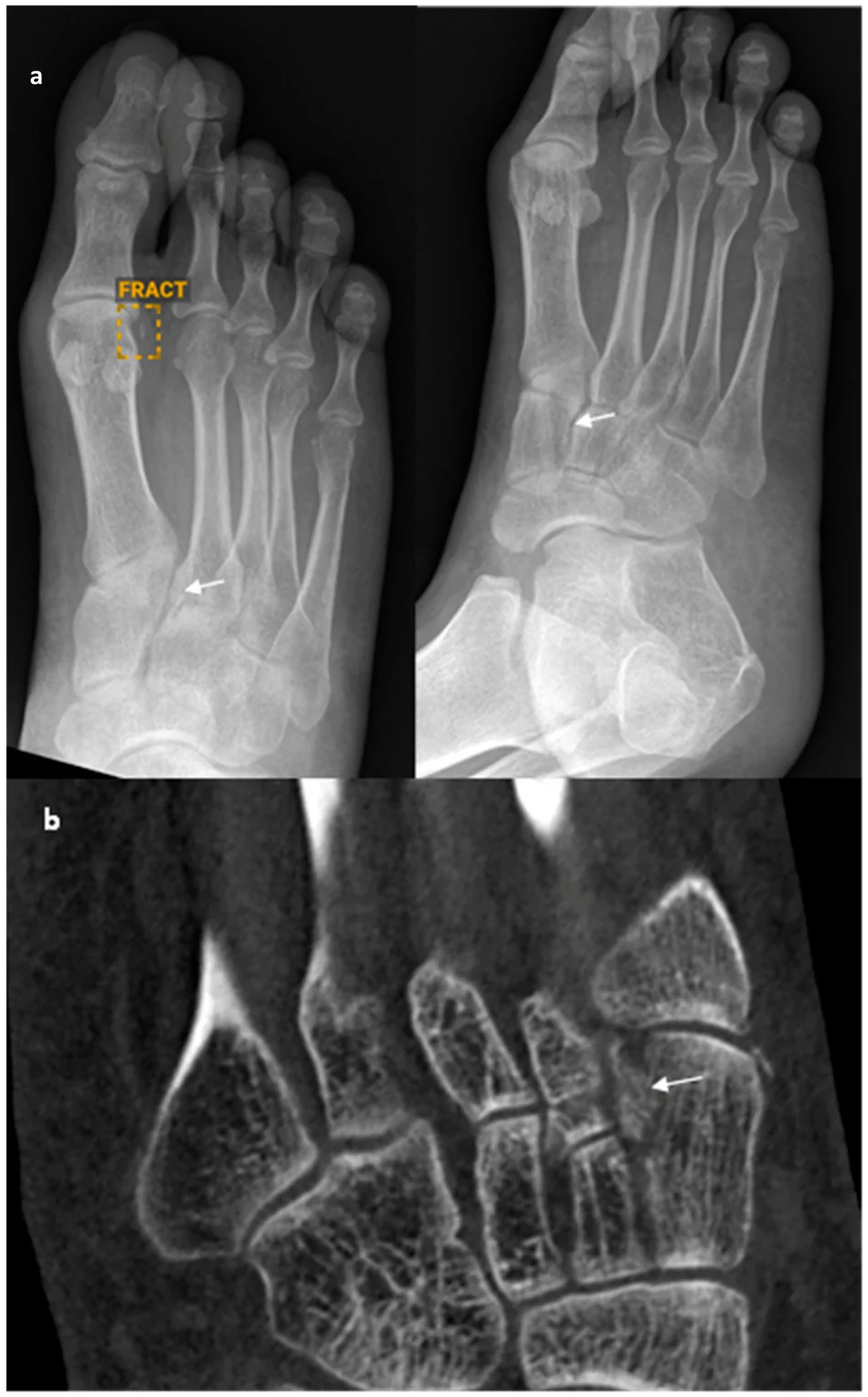
Discordant case of a Lisfranc joint injury. Autonomous AI did not detect the avulsed fragment in the metatarsal–cuneiform space (white arrow) on axial and oblique planes. Orange dotted bounding box is misinterpreted as a fracture (likely corticated post-traumatic fragment) (**a**). The radiologist detected the injury, and the diagnosis was subsequently confirmed on CBCT (white arrows) (**b**).

Regarding the prevalence of post-trauma injuries of Chopart and Lisfranc, we are aligned with literature findings as previously published (Abs B. et al. [1]). Early detection is important because these injuries, even when managed conservatively, can lead to complications such as post-traumatic arthritis if missed [4,5,6]; their impact on the healthcare system is therefore substantial. These injuries are notoriously difficult to detect due to overlapping anatomy and subtle cortical or ligamentous disruptions, as highlighted in the previous literature [1,2,3,4]. In the midfoot subgroups, radiologists were numerically more sensitive than standalone AI (Chopart 82.8% vs. 62.1%; Lisfranc 80.8% vs. 65.4%). Notably, 5 of the 26 Lisfranc injuries were purely ligamentous, without a cortical fracture; although BoneView can flag dislocations, it correctly identified only one of these five, which partly explains the lower crude AI sensitivity for Lisfranc. However, neither sensitivity comparison reached significance (exact McNemar *p* = 0.07 and *p* = 0.22), and only the Chopart accuracy comparison was significant (*p* = 0.039); these subgroups were underpowered (*n* = 29 and *n* = 26). The overall superiority of radiologists, significant for sensitivity, specificity and accuracy (all *p* < 0.0001) was driven mainly by the larger “other locations” category. Although the midfoot subgroups were underpowered, the significant overall gap and the numerically lower AI sensitivity at these sites are coherent with the underlying architecture of current AI systems, which are primarily trained on large heterogeneous datasets encompassing common fracture patterns, while rarer or anatomically intricate locations such as the midfoot remain underrepresented. Previous studies of the wrist, scaphoid, hand, ankle, and foot have consistently reported high specificity but more variable sensitivity, with overall diagnostic accuracy ranging from good to excellent [13,14]. They have shown that the AI detection rate drops in small or overlapping anatomical regions, where subtle cortical disruptions or dislocations are difficult to recognize even for trained radiologists (see Figure 2 and Figure 3) [13,14,15]. In these cases, AI misclassifications often arise from image projection variability, partial visualization, or low bone contrast. Such findings underscore the enduring importance of human pattern recognition, anatomical reasoning, and contextual clinical judgment, particularly in complex midfoot injuries that may appear radiographically subtle. In emergency trauma imaging, however, the primary concern is detection, avoiding missed fractures, making sensitivity the most critical parameter. These findings suggest that for anatomically complex midfoot injuries, AI shows promise but still requires further refinement.

Although our study evaluated AI in a fully autonomous setting, several investigations have demonstrated additional benefit when AI is used in assisted reading workflows [16,17,18]. At our institution, BoneView is not natively integrated into the radiologists’ reading environment; we therefore evaluated it in standalone mode, which also allowed us to characterize the algorithm’s intrinsic performance as a necessary first step before assessing it as a reading aid. When radiologists integrate AI outputs such as heatmaps into their interpretation, sensitivity can increase without a loss of specificity, sometimes shortening reading times and improving confidence [16,17,18]. However, this configuration was not tested in our study, and the potential gain remains to be verified in our population and workflow.

Our study also expands the limited evidence available for midfoot fractures, particularly at the Chopart and Lisfranc joints, where published data remain scarce. Previous works have highlighted that even with optimized projections, radiographs frequently miss up to 30–40% of fractures in these regions [1,2,3,4]. Cross-sectional imaging such as CT or cone-beam CT offers clear diagnostic advantages but is not systematically available in all emergency departments, which limits its routine use [5,6]. By evaluating AI performance against a mixed reference standard including CBCT and clinical follow-up, our work provides real-world insight into how such algorithms might behave under pragmatic emergency conditions rather than in highly curated datasets.

This study also offers several strengths. Radiograph interpretations by MSK radiologists were extracted directly from routine emergency workflow, providing real-world clinical performance rather than results generated under experimental reading conditions. The sample size was substantial and encompassed a broad spectrum of traumatic injuries across the foot and ankle. The explicit inclusion of Chopart and Lisfranc subgroups fills an important gap in the literature by providing novel diagnostic accuracy data for two anatomically complex and diagnostically challenging regions. Finally, the study design incorporated methodological safeguards, including anonymized image handling and a clearly defined hierarchical reference standard, which strengthen the transparency and reproducibility of the findings.

However, limitations must also be acknowledged. This was a single-center retrospective study conducted at a tertiary referral hospital, and the human comparison group consisted of radiologists specializing in musculoskeletal imaging. Their performance is likely superior to that of emergency physicians or general radiologists; consequently, the discrepancy between human interpreters and AI observed here might be smaller in community settings, and our results may not be directly generalizable to other centers, interpreter profiles, or acquisition protocols. Multicenter external validation is necessary before these results can be widely applied. Image readings were performed by several board-certified MSK radiologists from the same unit, ensuring real-world representativeness but introducing potential inter-reader variability. The hierarchical reference standard combined structured clinical follow-up with cross-sectional imaging when the radiograph was equivocal, and was established independently of the evaluated radiology report. This approach reflects real-world emergency practice, although a residual differential-verification effect cannot be entirely excluded. In addition, the study did not test AI-assisted readings or workflow integration; therefore, conclusions apply strictly to standalone AI performance. Because of the retrospective design, AI inference time and radiologists’ reporting time were not recorded. A prospective comparison of processing and reporting times would be needed to assess the tool’s efficiency in the clinical workflow. Finally, as with any evolving AI technology, software updates and dataset shifts may alter future performance, emphasizing the need for ongoing monitoring and revalidation [19,20,21,22].

## 5. Future Directions

Given the innovative approach of using AI to detect midfoot fractures, several lines of research are worth exploring. Future developments will likely focus on improving collaboration between humans and AI, with the latter helping radiologists enhance the accuracy, consistency, and efficiency of their workflows. Prospective, multicenter studies are needed to validate both standalone and AI-assisted performance across different centers, for different reader profiles, and according to different acquisition protocols. Specific development for complex anatomical regions such as the Chopart and Lisfranc joints, which remain underrepresented in training datasets, could improve the detection of subtle or purely ligamentous lesions, for which cross-sectional imaging or MRI remains necessary. Finally, interdisciplinary collaboration among radiologists, emergency physicians, and orthopedic surgeons will be essential to translate these tools into clinical practice.

## 6. Conclusions

In this single-center study of 701 foot and ankle examinations, including 29 Chopart and 26 Lisfranc injuries, standalone AI achieved high overall diagnostic performance but was significantly less sensitive, specific and accurate than expert musculoskeletal radiologists (all *p* < 0.0001); in the midfoot subgroups, radiologists were numerically more sensitive, although these underpowered comparisons did not reach significance. These findings underline both the promise and the current limitations of autonomous AI: while it provides rapid and consistent fracture screening, it does not yet match expert performance. In its present form, AI should therefore be regarded as an assistive adjunct to expert radiologists, not a replacement for them. Continued progress in this field will likely come from further refinement of AI algorithms and from prospective evaluation of how such tools can be integrated into clinical workflows to support diagnostic consistency, efficiency, and timely decision-making in emergency settings.

## Figures and Tables

**Table 1:** Confusion matrices of the radiologist and standalone AI against the reference standard, overall and by subgroup.

Reader/Group	TP	FP	FN	TN	Total
Overall (*n* = 701)					
Radiologist	268	17	51	365	701
AI (BoneView)	237	65	82	317	701
Chopart (29 positive)					
Radiologist	24	2	5	670	701
AI (BoneView)	18	3	11	669	701
Lisfranc (26 positive)					
Radiologist	21	0	5	675	701
AI (BoneView)	17	1	9	674	701

TP, true positive; FP, false positive; FN, false negative; TN, true negative. Counts are given against the hierarchical reference standard (structured clinical follow-up complemented by cross-sectional imaging when the radiograph was equivocal).

**Table 2:** Overall diagnostic performance (all foot/ankle fractures).

Method	Sensitivity % (95% CI)	Specificity % (95% CI)	PPV % (95% CI)	NPV % (95% CI)	Likelihood Ratio +	Likelihood Ratio −	Accuracy % (95% CI)
AI (BoneView)	74.3 (69.2–78.8)	83.0 (78.9–86.4)	78.5 (73.5–82.7)	79.4 (75.2–83.1)	4.37 (3.47–5.50)	0.31 (0.26–0.38)	79.0 (75.9–81.9)
Radiologist	84.0 (79.6–87.6)	95.5 (93.0–97.2)	94.0 (90.7–96.2)	87.7 (84.2–90.6)	18.67 (11.83–30.12)	0.17 (0.13–0.22)	90.3 (87.9–92.3)

Agreement (AI vs. Radiologist): Cohen’s κ = 0.65. McNemar (global): *p* < 0.0001 for sensitivity, specificity and accuracy. Metrics computed against the hierarchical reference standard.

**Table 3:** Diagnostic performance for Chopart (N = 701).

Method	Sensitivity % (95% CI)	Specificity % (95% CI)	PPV % (95% CI)	NPV % (95% CI)	Accuracy % (95% CI)
AI (BoneView)	62.1 (44.0–77.3)	99.6 (98.7–99.8)	85.7 (65.4–95.0)	98.4 (97.1–99.1)	98.0 (96.7–98.8)
Radiologist	82.8 (65.5–92.4)	99.7 (98.9–99.9)	92.3 (75.9–97.9)	99.3 (98.3–99.7)	99.0 (98.0–99.5)

Agreement (AI vs. Radiologist): Cohen’s κ = 0.80. exact McNemar: sensitivity *p* = 0.07; specificity *p* = 1.0 accuracy *p* = 0.039.

**Table 4:** Diagnostic performance for Lisfranc (N = 701).

Method	Sensitivity % (95% CI)	Specificity % (95% CI)	PPV % (95% CI)	NPV % (95% CI)	Accuracy % (95% CI)
AI (BoneView)	65.4 (46.2–80.6)	99.9 (99.2–100.0)	94.4 (74.2–99.0)	98.7 (97.5–99.3)	98.6 (97.4–99.2)
Radiologist	80.8 (62.1–91.5)	100.0 (99.4–100.0)	100.0 (84.5–100.0)	99.3 (98.3–99.7)	99.3 (98.3–99.7)

Agreement (AI vs. Radiologist): Cohen’s κ = 0.82. Exact McNemar sensitivity: *p* = 0.22; specificity *p* = 1.0 accuracy *p* = 0.125.

## Data Availability

The datasets generated and/or analyzed during the current study are not publicly available due to institutional and ethical restrictions but are available from the corresponding author on reasonable request.
